# Temporal changes in monocyte subsets and cytokine production capacity of mononuclear cells following myocardial infarction

**DOI:** 10.1016/j.jmccpl.2026.100876

**Published:** 2026-10-09

**Authors:** Jonathan Los, Ilse H. Hol, Frans B. Mensink, Malin Östman, Aysun Cetinyurek-Yavuz, Jan H. Cornel, Robert-Jan M. van Geuns, Niels P. Riksen, Saloua El Messaoudi

**Affiliations:** a Department of Cardiology, Radboud University Medical Centre, Nijmegen, the Netherlands; b Department of Internal Medicine, Radboud University Medical Centre, Nijmegen, the Netherlands; c Department of IQ Health, Radboud University Medical Centre, Nijmegen, the Netherlands; d Department of Cardiology, Northwest Clinics, Alkmaar, the Netherlands; e Dutch Network for Cardiovascular Research (WCN), Utrecht, the Netherlands

**Keywords:** Myocardial infarction, Monocyte phenotype, Inflammation, Peripheral blood mononuclear cells, Biomarkers

## Abstract

Following myocardial infarction (MI), recruited monocytes play an essential role in tissue repair and scar formation. However, the temporal changes in circulating monocyte subsets and their functional characteristics remain incompletely understood. We investigated systemic inflammation and circulating monocyte heterogeneity in 72 patients during the first 12 weeks after MI. Systemic inflammation was markedly increased immediately after MI, as reflected by significant elevated C-reactive protein and interleukin-6 (IL-6) levels. During follow-up, the proportion of classical monocytes decreased, whereas the relative abundance of non-classical monocytes increased. In the acute phase, cluster of differentiation 11b expression on intermediate monocytes was elevated, while peripheral blood mononuclear cells produced lower levels of the anti-inflammatory cytokines IL-10 and IL-1Ra. Together, the results support the presence of a relatively more inflammatory monocyte profile during the acute phase of MI.

## Highlights

• During the early weeks after myocardial infarction (MI), classical monocytes decrease and non-classical monocytes increase in relative abundance.
• Immediately post-MI, intermediate monocytes exhibit increased expression of CD11b, and mononuclear cells produce lower levels of anti-inflammatory cytokines.
• These findings indicate a relatively more pro-inflammatory monocyte profile during the acute phase of MI.

## Graphical abstract

Abbreviations: CD, cluster of differentiation; IL-10, interleukin-10; IL-1Ra, interleukin-1 receptor antagonist; PBMCs, peripheral blood mononuclear cells

**Figure:**
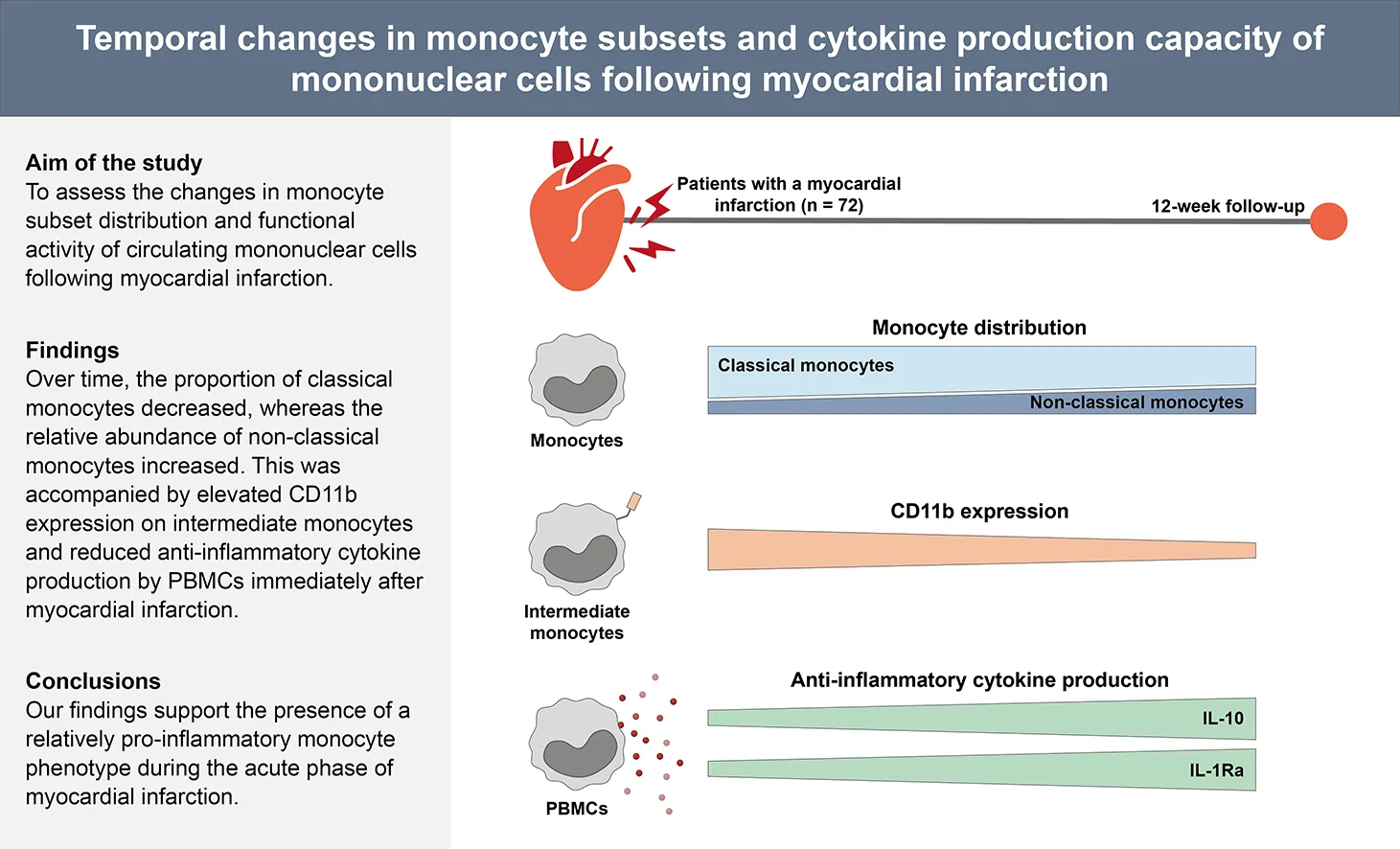

## Abbreviations

**Table:** 

CD	Cluster of differentiation
cMo	Classical monocyte
CRP	C-reactive protein
IL-1Ra	Interleukin-1 receptor antagonist
IL-6	Interleukin-6
intMo	Intermediate monocyte
LPS	Lipopolysaccharide
MI	Myocardial infarction
ncMo	Non-classical monocyte
NSTEMI	Non-ST-elevation myocardial infarction
PBMC	Peripheral blood mononuclear cell
STEMI	ST-elevation myocardial infarction
TNFα	Tumour necrosis factor α

## 1 Introduction

A myocardial infarction (MI) induces an acute surge in inflammatory activity, characterized by rapid recruitment of circulating monocytes to the damaged myocardium to orchestrate tissue repair [1], [2]. Timely resolution of local inflammation appears to be a prerequisite for adequate myocardial healing, whereas excessive inflammatory activity may disrupt regenerative processes [1], [2], [3], [4], [5]. Monocytes comprise heterogeneous populations that are thought to exert distinct functions in myocardial healing [6], [7]. Murine studies demonstrated two distinct phases in circulating monocytes and myocardial monocyte recruitment [8]. The first phase is dominated by inflammatory Ly-6c^high^ monocytes, followed by Ly-6c^low^ monocytes which have attenuated inflammatory properties [8]. Human studies support the biphasic pattern, with an initial rise in circulating classical monocytes (cMo, the human equivalent of Ly-6c^high^ monocytes) followed by an increase in non-classical monocytes (ncMo, the human equivalent of Ly-6c^low^ monocytes) [4], [6], [9]. However, the relationship between these temporal changes in monocyte heterogeneity and their functional roles remains incompletely understood. To this end, we examined the subset distribution and functional activity of circulating monocytes in patients following MI.

## 2 Patients and methods

This study includes 72 patients admitted for acute MI to the Radboud University Medical Centre, Nijmegen, the Netherlands who were recruited for the FITTER trial (NCT04141579) [10]. In this trial, patients were randomized to receive evolocumab or placebo in addition to high-intensity statin therapy to assess the short-term effects of lipid-lowering therapy on non-culprit stenosis severity. For the present analysis, the treatment groups were pooled after confirming the absence of any between-group differences in inflammatory parameters. Alongside randomisation to additional lipid-lowering therapy, patients were treated according to contemporary guidelines [11]. Ethical approval was given by the Dutch Ethical Review Board (METC Oost Nederland) and the study was conducted in full accordance with the principles of the “Declaration of Helsinki.” All participants provided written informed consent. The patients underwent repeated blood sampling after successful percutaneous coronary intervention of the culprit artery and at 4- and 12-week follow-up. Patients with an active malignancy were excluded from the analysis. In addition, outcome data from samples in patients with evident infections or vaccinations occurring within one week prior to blood collection were not included.

Plasma samples were obtained at every time point, whereas peripheral blood mononuclear cells (PBMCs) were isolated in the early post-MI period and at week 12 from freshly collected blood using BD Vacutainer® dipotassium ethylene diamine tetra-acetic acid (K2-EDTA) tubes (BD Biosciences, San Jose, USA). Samples were stored at −150 °C for subsequent simultaneous assessment of systemic inflammatory biomarkers, PBMC stimulation, and flow cytometry analysis (**Supplemental Methods**).

C-reactive protein (CRP) and interleukin-6 (IL-6) were quantified in plasma using DuoSet ELISA kits and Quantikine ELISA kits (Bio-Techne/R&D Systems, Minneapolis, USA), respectively. We performed flow cytometry analysis (**Supplementary fig. 1**) on thawed PBMCs using mouse monoclonal antibodies against cluster of differentiation (CD) 14 and CD16, to assess the three monocyte subsets (CD14^++^CD16^−^ cMo, CD14^++^CD16^+^ intermediate monocytes [intMo], and CD14^+^CD16^++^ ncMo), according to current guidelines [12]. CD11b was included as important adhesion and signalling marker [13]. In addition, thawed PBMCs were stimulated for 24 h with 10 ng/mL lipopolysaccharide (LPS) from *Escherichia coli* for Toll-like receptor-4 stimulation (Sigma-Aldrich, St. Louis, USA). In the supernatants, we measured the pro-inflammatory cytokines tumour necrosis factor α (TNFα), IL-1β, and IL-6, and the anti-inflammatory cytokines IL-10 and interleukin-1 receptor antagonist (IL-1Ra) with DuoSet ELISA kits (Bio-Techne R&D Systems, Minneapolis, USA).

Values are reported as mean ± SD, median (25th percentile - 75th percentile), or count (%). First, to ensure that there were no differences between the treatment groups, we conducted linear mixed models, including time, treatment group, and their interaction as fixed effects, with a random intercept for patient to account for within-subject correlation. Subsequently, we continued with the assessment of overall changes over time. For CRP and IL-6, linear mixed models were used to assess temporal changes, with time included as fixed effect and patient as random effect. Changes in monocyte subsets, CD11b expression, and cytokine production capacity were evaluated using either the Wilcoxon signed-rank test or paired *t*-tests, as appropriate. Differences between patients with non-ST-elevation myocardial infarction (NSTEMI) and those with ST-elevation myocardial infarction (STEMI) patients were also assessed using a linear mixed model approach, including an interaction term between time and MI type. In addition, differences in time from symptom onset to blood sampling between STEMI and NSTEMI patients were assessed using the Mann–Whitney *U* test, while associations between time from symptom onset to blood sampling immediately post-MI and monocyte phenotype and PBMC cytokine production capacity were explored using Spearman's rank correlation. All reported *p*-values are 2-sided. To control for multiple testing, *p*-values were adjusted per analysis group using the Benjamini–Hochberg procedure with a false discovery rate of 5%. All statistical analyses were performed using R-studio version 4.4.2 (R Foundation for Statistical Computing, Vienna, Austria).

## 3 Results

In total, 72 patients (mean age 65.1 ± 9.1 years, 19.4% female) were included in the study (Table 1). The majority of patients presented with NSTEMI (66.7%) and median maximum creatine kinase level was 413 U/L (158 to 1302). Median time between onset of symptoms and baseline blood withdrawal was 1.2 days (0.6 to 2.9), with no significant difference between STEMI (1.2 days [0.2 to 2.9]) and NSTEMI (1.4 days [0.7 to 3.0]) patients (*P* = 0.264). Eventually, data from 71 patients for monocyte phenotyping and 65 patients for PBMC cytokine production capacity were included in the analyses (**Supplementary fig. 2**).

**Table 1:** Baseline characteristics (*n* = 72).

	Study population
Demographics	
Age – years	65.1 ± 9.1
Sex, female	14 (19.4%)

Cardiovascular risk factors	
BMI – kg/m^2^	27.2 ± 3.4
Hypertension	26 (36.1%)
Dyslipidaemia	27 (37.5%)
Family history of premature CAD	34 (47.2%)
Current smoking	20 (27.8%)
Diabetes mellitus	7 (9.7%)

Medical history	
Stroke or transient ischemic attack	5 (6.9%)
Peripheral artery disease	2 (2.8%)
Prior myocardial infarction	9 (12.5%)
Prior percutaneous coronary intervention	12 (16.7%)

Medication at admission	
Aspirin	15 (20.8%)
ADPRI (ticagrelor/clopidogrel/prasugrel)	5 (6.9%)
Statins	17 (23.6%)
ACE inhibitor	7 (9.7%)
ARB	2 (2.8%)
Beta-blocker	14 (19.4%)

Clinical presentation	
STEMI	24 (33.3%)
NSTEMI	48 (66.7%)
Maximum creatine kinase level – U/L⁎	413 (158–1302)

Values are presented as mean ± SD, median (25th – 75th percentile) or count (%).

Abbreviations: ACE, angiotensin converting enzyme; ADPRI, adenosine diphosphate receptor inhibitor; ARB, angiotensin receptor blocker; BMI, body mass index (calculated as weight in kilograms divided by height in meters squared); CAD, coronary artery disease; NSTEMI, non-ST-segment elevation myocardial infarction; STEMI, ST-segment elevation myocardial infarction.

⁎ Creatine kinase levels were missing for one patient.

There were no differences in change observed for any of the parameters between the treatment groups (*i.e.* evolocumab *vs.* placebo), allowing for the pooling of data to assess overall changes over time (**Supplementary tables 1–3**). Immediately following MI, CRP and IL-6 were higher compared with week 4 and week 12 (all P_adj_ < 0.001) (Fig. 1A). No significant differences in CRP or IL-6 concentrations were observed between week 4 and week 12, indicating that systemic inflammation had returned to background levels beyond week 4 (Fig. 1A). With respect to monocyte subsets, cMo were relatively more abundant at the early post-MI timepoint compared with follow-up (79.3% [74.8 to 83.5] *vs.* 76.5% [71.8 to 81.7]; P_adj_ = 0.025), whereas the percentage of ncMo increased over time (5.2% [3.1 to 6.9] *vs.* 6.2% [4.7 to 8.9]; P_adj_ = 0.001) (Fig. 1B). In addition, CD11b expression on intMo was significantly higher in the early post-MI period (P_adj_ = 0.011) (Fig. 1B). At 12-week follow-up, PBMCs produced significantly higher amounts of the anti-inflammatory cytokines IL-10 (P_adj_ = 0.004) and IL-1Ra (P_adj_ = 0.004) upon LPS stimulation, whereas the production of the pro-inflammatory cytokines TNFα (P_adj_ = 0.734), IL-1β (P_adj_ = 0.358), and IL-6 (P_adj_ = 0.085) was similar compared to early post-MI (Fig. 1C). Changes in monocyte phenotype were generally larger in STEMI patients, although only the increase in ncMo abundance over time differed significantly (between-group difference, 2.7% [95%CI, 0.9 to 4.6]; P_adj_ = 0.024) (**Supplementary table 4**). There was no difference in the change in PBMC cytokine production between STEMI and NSTEMI patients (**Supplementary table 5**). Time from symptom onset to blood sampling immediately post-MI was negatively correlated with the relative abundance of cMo (ρ = −0.37, P_adj_ = 0.004) and positively correlated with the relative abundance of intMo (ρ = 0.51, P_adj_ < 0.001). No significant associations were observed between time from symptom onset and the relative proportion of ncMo, CD11b expression, or PBMC cytokine production capacity during the acute phase.

**Fig. 1:**
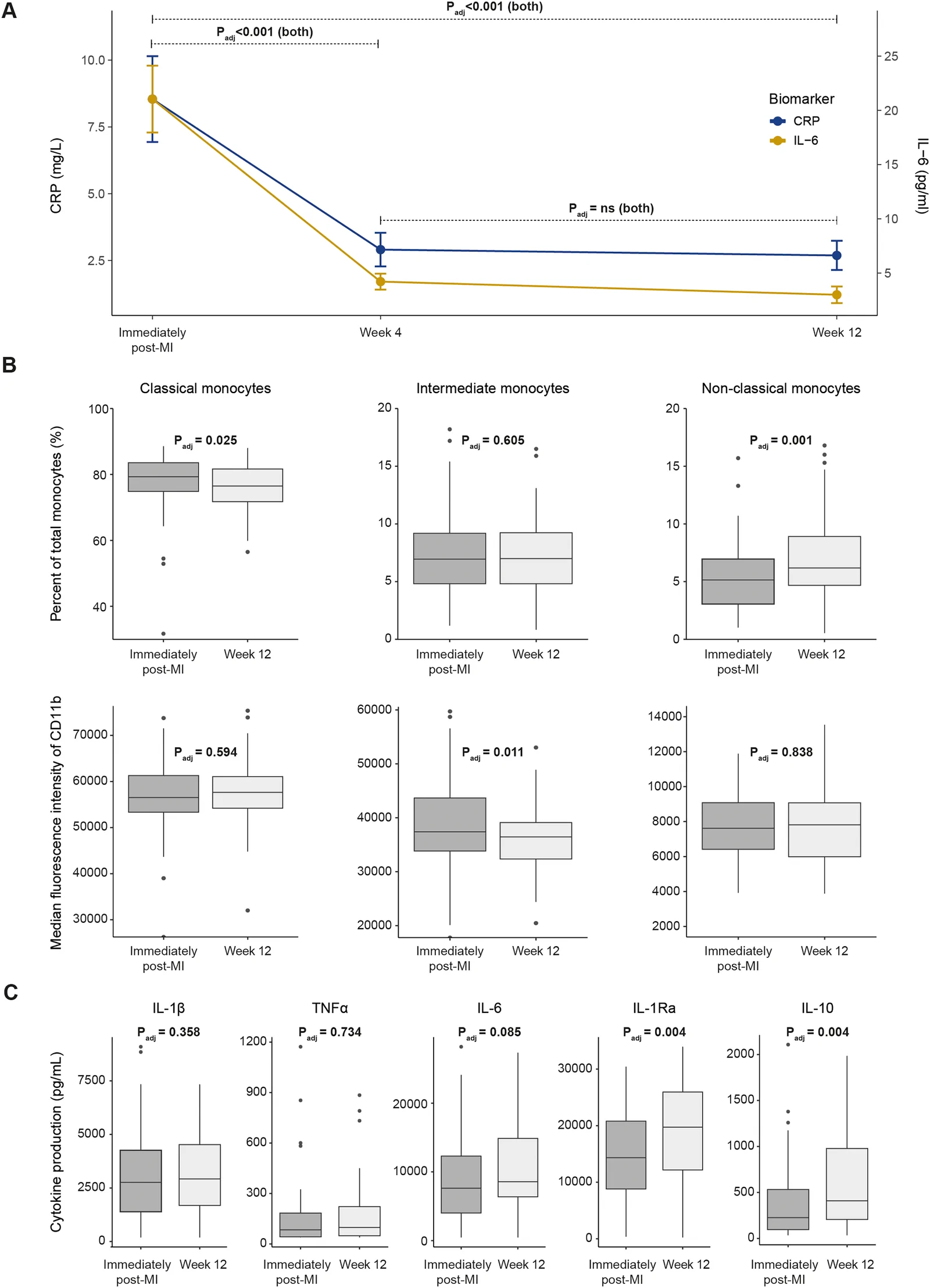
Monocyte heterogeneity and change in cytokine production capacity of mononuclear cells following myocardial infarction (A) Temporal trajectories of systemic inflammatory biomarkers (*n* = 72 patients), assessed using linear mixed models with time as a fixed effect and patient as a random effect. Values are presented as mean ± standard error of the mean. One value was excluded because of a vaccination a few days before blood sampling at week 4 (*n* = 71). (B) Boxplots of the changes in major monocyte subset populations and monocyte CD11b expression (*n* = 71 patients), assessed using the Wilcoxon signed-rank test or paired *t*-test, as appropriate. (C) Boxplots of the changes in cytokine production capacity of peripheral blood mononuclear cells (*n* = 65 patients), assessed using the Wilcoxon signed-rank test. All reported *p*-values were adjusted for multiple testing within each analysis using the Benjamini–Hochberg procedure, with a false discovery rate of 5% (adjusted *P* < 0.05 considered statistically significant). Abbreviations: CD, cluster of differentiation; CRP, C-reactive protein; IL, interleukin; IL-1Ra, interleukin-1 receptor antagonist; MI, myocardial infarction; ns, non-significant; TNFα, tumour necrosis factor α.

## 4 Discussion

Our findings demonstrate distinct differences in monocyte subset distribution and PBMC cytokine production capacity between the early post-MI period and 12-week follow-up, at which point systemic inflammation had resolved. Specifically, the percentage of cMo was higher in the acute phase, while ncMo were more prevalent at follow-up. Importantly, this shift was associated with lower anti-inflammatory cytokine production by PBMCs in the early post-MI period. Additionally, intMo exhibited higher CD11b expression immediately post-MI. Of interest, changes in monocyte phenotype were more pronounced in STEMI patients. Taken together, these findings indicate a relatively more pro-inflammatory monocyte profile during the acute phase of MI.

Together with neutrophils, monocytes are the first leukocyte population to accumulate in the injured myocardium [5], [14]. Their biological aim is to promote tissue repair by removing necrotic debris, stimulating angiogenesis, and supporting extracellular matrix deposition, thereby facilitating scar formation [2], [15]. After a MI, there is a rapid release of cMo from the bone marrow and possibly the spleen into the circulation [16], [17]. Only a few studies investigated shifts in monocyte subset in the context of MI in humans. *Tsujioka et al* demonstrated a biphasic increase in circulating monocyte subsets, with cMo peaking at 2.6 days, followed by a peak in CD16^+^ monocytes (intMo and ncMo) at day 5 [4]. *Tapp et al* found a similar pattern and increased percentage of ncMo at 30 days following MI [9]. In addition, post-mortem studies showed a spatiotemporal pattern of myocardial cMo accumulation, with preferential localization to the infarct border zone during the inflammatory phase, followed by infiltration of the necrotic core by cMo and CD16^+^ monocytes during the later phase [17]. Our findings align with the transient expansion of cMo induced by acute inflammation followed by a later increase in CD16^+^ monocytes, as supported by a clear post-MI shift from cMo to ncMo and the negative correlation between time from symptom onset to first blood sampling and the relative abundance of cMo. This negative correlation also suggests that the decrease in cMo after the initial surge starts early. The more pronounced changes observed in STEMI patients may reflect greater mobilisation of circulating monocytes in response to a more abrupt inflammatory stimulus resulting from acute coronary occlusion compared with NSTEMI patients. In addition to major monocyte subset heterogeneity, we assessed monocyte CD11b expression, which is a surface glycoprotein facilitating cell adhesion, migration, and phagocytosis [13]. In the acute post-MI period, intMo showed higher CD11b-expression compared to follow up. This potentially facilitates increased adhesion and migration of intMo to the infarcted myocardium [6].

Studies on the inflammatory function of circulating monocytes immediately post-MI remains scarce. It is thought that distinct monocyte subclasses serve different functions [6]. Among monocyte subsets, cMo and intMo exhibit high phagocytic capacity and secrete pro-inflammatory cytokines, whereas ncMo are believed to contribute to inflammation resolution, debris clearance, and vascular patrolling [6]. In our study, the lower production of the anti-inflammatory cytokines IL-10 and IL-1Ra by PBMCs immediately post-MI supports the pro-inflammatory role of cMo, while indicating that ncMo may contribute to active resolution mechanisms by a higher production of anti-inflammatory cytokines. In line with our results, v*an Haelst et al* found that monocytes isolated from patients with ACS immediately upon admission produced less IL-10 after LPS simulation compared with healthy controls [18]. *Blokland et al* investigated differential gene expression in PBMCs from patients with STEMI [19]. Compared with healthy controls, monocytes exhibited the largest number of differentially expressed genes involved in both pro- and anti-inflammatory pathways during the acute phase of MI, highlighting their role in post-infarction remodelling [19]. Furthermore, enrichment of the anti-inflammatory IL-10 signalling pathway increased over time [19]. Collectively, circulating leukocyte function appears to be relatively more pro-inflammatory during the acute phase of MI, gradually shifting toward a less inflammatory profile over time.

### 4.1 Limitations

Unfortunately, in humans, it is impossible to collect blood samples before an acute MI, which would have been valuable for assessing baseline levels. In addition, no serial blood sampling was performed in the first hours or days following MI, restricting the ability to evaluate changes in the immediate window after the coronary event. Also, data on absolute circulating leukocyte counts were unavailable, and cytokine production was assessed in PBMCs rather than isolated monocytes, precluding attribution of the observed changes specifically to monocytes. Furthermore, cryopreserved samples were used for flow cytometry analysis and to assess cytokine production. Nonetheless, an average of 95.3% of cells across all our samples survived freezing and thawing. Also, previous analyses have demonstrated that cryopreserved cells generally compare well with fresh cells [20].

## 5 Conclusion

During the first few weeks following MI, the relative abundance of cMo decreases, whereas that of ncMo increases. This was associated with reduced anti-inflammatory cytokine production by PBMCs and increased CD11b expression on intMo during the acute phase. Collectively, these observations support a relatively heightened inflammatory monocyte phenotype during the acute phase of MI. Our findings are essential for potential future therapeutic strategies that modulate immune function.

## CRediT authorship contribution statement

**Jonathan Los:** Writing – original draft, Visualization, Project administration, Methodology, Investigation, Formal analysis, Data curation, Conceptualization. **Ilse H. Hol:** Writing – review & editing, Methodology, Investigation, Formal analysis. **Frans B. Mensink:** Writing – review & editing, Investigation, Data curation. **Malin Östman:** Writing – review & editing, Methodology, Investigation. **Aysun Cetinyurek-Yavuz:** Writing – review & editing, Formal analysis. **Jan H. Cornel:** Writing – review & editing, Conceptualization. **Robert-Jan M. van Geuns:** Writing – review & editing, Resources, Project administration, Funding acquisition, Conceptualization. **Niels P. Riksen:** Writing – review & editing, Resources, Conceptualization. **Saloua El Messaoudi:** Writing – review & editing, Conceptualization.

## Funding sources

The Radboud University Medical Centre received funding by Amgen Europe B.V., Infraredx inc., and Health Holland to execute this study. The funding providers were not involved in study protocol design, data acquisition, or data analysis.

## Declaration of competing interest

Niels P Riksen is financially supported by a consortium grant of the Dutch Heart Foundation (AtheroNeth; 01–001–2024–0601) and a Vici grant of the Netherlands Organisation for Health Research and Development (ZonMw; 09150182410037).

Robert-Jan M van Geuns reports consulting and speaker fees from Abbott Vascular and Astra Zeneca, and received institutional research grants from Amgen, InfraRedx, Astra Zeneca, and Sanofi.

The remaining authors report no relationships that could be construed as a conflict of interest.

## Data Availability

The data that support the findings of this study are available from the corresponding author upon reasonable request.
