# Supplementary material for: Temporal changes in monocyte subsets and cytokine production capacity of mononuclear cells following myocardial infarction
Source: J Mol Cell Cardiol Plus. 2026 Oct 9;18:100876. doi: 10.1016/j.jmccpl.2026.100876 (PMC13720828; doi:10.1016/j.jmccpl.2026.100876)
Supplement: Supplementary file 1 [file mmc1.docx]

## Supplemental methods

### Laboratory experiments

Freshly collected blood samples were obtained using BD Vacutainer® dipotassium Ethylene Diamine Tetra Acetic acid (K2-EDTA) tubes (BD Biosciences, San Jose, USA). Plasma was acquired from the EDTA tubes after centrifugation (2744g for 10 min at room temperature). Peripheral blood mononuclear cells (PBMCs) were isolated using the Ficoll-paque™ Plus density gradient centrifugation (Cytiva, Life Sciences, Marlborough, USA) method. The PBMCs were resuspended in Roswell Park Memorial institute 1640 Dutch modified culture medium (RPMI, Life Technologies, Carlsbad, USA), supplemented with 2 mmol/L GlutaMAX (Gibco, Waltham, USA), 1 mmol/L pyruvate (Gibco, Waltham, USA), and 50 μg/mL gentamycin (Centrafarm, Breda, the Netherlands). Gibco Recovery Cell Culture Freezing Medium (Thermo Fisher Scientific, Paisley, UK) was used to freeze the cells. Plasma and PBMCs were stored at −150 ◦C for later inflammatory biomarker assessment, PBMC stimulation and flow cytometry analysis.

In plasma samples, C-reactive protein was quantified using DuoSet ELISA kits (Bio-Techne/R&D Systems, Minneapolis, USA), and interleukin-6 (IL-6) was measured using the Quantikine ELISA kits (Bio-Techne/R&D Systems, Minneapolis, USA), according to the manufacturer's instructions.

Frozen PBMCs were gradually thawed and washed with RPMI supplemented with heat-inactivated foetal calf serum (Capricorn Scientific, Ebsdorfergrund, Germany). Cell count and viability check were assessed manually. An average of 95.3% of cells across all our samples survived freezing and thawing. To evaluate cytokine production capacity, 1 × 10^5^ PBMCs were stimulated in duplicate for 24 h in round-bottom 96-well plates (Corning, New York, USA) and incubated with RPMI (negative control) and 10 ng/mL lipopolysaccharide from Escherichia coli for Toll-like receptor−4 stimulation (Sigma-Aldrich, St. Louis, USA). After incubation for 24 h at 37 °C with 5% CO2, supernatants were collected after centrifugation of the plates (359g for 8 min at room temperature) and stored at -20 °C until cytokine measurement. From the separated supernatants, the secreted cytokines tumour necrosis factor α (TNFα), IL-1β, IL-6, IL-10 and IL-1Ra were measured using commercially available DuoSet ELISA kits (Bio-Techne R&D Systems, Minneapolis, USA). Although myocardial infarction may induce spontaneous cytokine production through the release of damage-associated molecular patterns, elevated cytokine concentrations in unstimulated controls may also result from experimental factors, such as potential endotoxin or sample contamination. Therefore, patients were excluded if cytokine concentrations in unstimulated controls exceeded twice the lower limit of detection for at least two of three cytokines (TNF-α, IL-1β, and IL-6) at either time point. This resulted in the exclusion of 7 patients.

Flow cytometry analysis was performed to quantify monocyte subsets and cluster of differentiation (CD) 11b expression. Therefore, 1 × 10^5^ PBMCs were washed with flow cytometry staining buffer (phosphate-buffered saline with albumin and EDTA [PBA-E]: 1% bovine serum albumin [BSA] in phosphate-buffered saline with 2mM EDTA). Cells were incubated with 10 µl Human TruStain FcX (Biolegend, San Diego, USA) Fc block for 10 minutes. Subsequently, the cells were stained with 2.5 µL of each mouse monoclonal antibody specific for CD14 (anti-human CD14 PE-Cy7 61D3 [eBioscience, San Diego, USA]), CD16 (FITC anti-human CD16 antibody [Biolegend, San Diego, USA]), and CD11b (Pacific Blue™ anti-human CD11b antibody [Biolegend, San Diego, USA]), mixed with 12.5 µl of PBA-E to prevent cell clumping. After 20 min incubation at room temperature, cells were washed and resuspended in 150 µl of PBA-E. Helix NiR viability dye (Biolegend, San Diego, USA) was added for 15 min at room temperature in the dark, to be able to discriminate live- and dead cells. Cell populations and expression of CD11b were measured using a CytoFlex cytometer (Beckman Coulter, Brea, USA), which underwent daily quality control. For each measurement, an unstained control sample was included to assess autofluorescence.

Flow cytometry analysis was performed using OMIQ flow analysis software and a manual gating strategy (**Supplementary figure 1**). First, single cells were gated using the forward scatter height (FSC-H) vs. forward scatter area (FSC-A). Subsequently, the lymphocyte and monocyte populations were gated using FSC-A vs. side scatter area (SSC-A). Live cells were selected using a Helix NiR vs. SSC-A plot. A new FSC-A vs. SSC-A plot was created, which showed the refined lymphocyte and monocyte populations. Next, monocyte subsets (classical monocytes, intermediate monocytes, and non-classical monocytes) were identified using a CD14 vs. CD16 density plot. For each monocyte subset, a CD11b histogram was generated to assess and select CD11b-positive cells.

## Supplementary figure 1. Flow cytometry gating strategy

A manual gating strategy was employed to identify monocyte subsets in thawed PBMCs using flow cytometry. (A) Separation of single cells from doublets. (B) Gating of lymphocyte and monocyte populations based on forward and side scatter, along with the exclusion of granulocytes and debris. (C) Selection of live cells using Helix Nir viability dye. (D) Identification of the refined monocyte population based on monocyte scatter properties. (E) Monocyte subsets (classical monocytes, intermediate monocytes, and non-classical monocytes) were defined using a CD14 and CD16 density plot and were expressed as percentage of the refined monocyte population. (F) Finally, CD11b expression was gated for CD11b-positive cells within each individual subset.

Abbreviations: CD, cluster of differentiation; FSH-A, forward scatter area; FSH-H, forward scatter height; PBMC, peripheral blood mononuclear cells; SSC-A, side scatter area

## Supplementary figure 2. Study flowchart

Abbreviations: PBMC, peripheral blood mononuclear cells

## Supplementary table 1. Change in inflammatory biomarkers by treatment group (n = 72)

| **Biomarker** | **Timepoint** | **Evolocumab (n= 35)**  **mean±SD** | **Placebo (n = 37)**  **mean±SD** | **P_adj_ value** |
| --- | --- | --- | --- | --- |
| CRP | Baseline | 10.7±15.7 | 6.5±11.2 | 0.272 |
|  | Week 4 | 2.8±4.0* | 3.0±6.3 |  |
|  | Week 12 | 2.1±2.4 | 3.3±6.0 |  |
| IL-6 | Baseline | 19.1±23.0 | 22.9±28.9 | 0.692 |
|  | Week 4 | 3.4±4.6* | 5.0±7.4 |  |
|  | Week 12 | 3.2±6.8 | 2.8±6.3 |  |

Differences in change between the treatment groups (evolocumab-treated patients, n = 34; placebo-treated patients, n = 37) were assessed using linear mixed-effects models, including time, treatment group, and their interaction as fixed effects, with a random intercept for participant. P-values were adjusted for multiple testing using the Benjamini–Hochberg procedure with a false discovery rate of 5%.

* One value was excluded because of a vaccination a few days before blood sampling at week 4 (n = 34).

Abbreviations: CRP, C-reactive protein; IL-6, interleukin-6

## Supplementary table 2. Change in monocyte phenotype by treatment group (n = 71)

| **Parameter** | **Treatment group** | **Baseline**  **mean±SD** | **Follow-up**  **mean±SD** | **Mean change**  **(95% CI)** | **Mean difference in change**  **(95%CI) vs. placebo** | **P_adj_ value** |
| --- | --- | --- | --- | --- | --- | --- |
| Classical monocytes, % | Evolocumab (n = 34) | 75.5±11.7 | 75.2±6.9 | -0.2 (-3.6 to 3.1) | 2.3 (-2.4 to 7.0) | 0.693 |
|  | Placebo (n = 37) | 79.4±5.8 | 76.9±7.2 | -2.5 (-5.8 to 0.7) |  |  |
| Intermediate monocytes, % | Evolocumab (n = 34) | 7.9±3.8 | 8.0±3.0 | 0.1 (-1.0 to 1.3) | 0.1 (-1.5 to 1.7) | 0.925 |
|  | Placebo (n = 37) | 6.4±3.0 | 6.5±3.4 | 0.1 (-1.1 to 1.2) |  |  |
| Non-classical monocytes, % | Evolocumab (n = 34) | 5.5±3.1 | 8.1±3.6 | 2.6 (1.2 to 3.9) | 1.4 (-0.4 to 3.2) | 0.693 |
|  | Placebo (n = 37) | 5.0±2.5 | 6.2±3.6 | 1.2 (-0.1 to 2.4) |  |  |
| CD11b – classical monocytes, MFI | Evolocumab (n = 34) | 56964±8477 | 57428±7664 | 463 (-1372 to 2299) | -147 (-2689 to 2395) | 0.925 |
|  | Placebo (n = 37) | 57109±7104 | 57719±6335 | 610 (-1149 to 2370) |  |  |
| CD11b – intermediate monocytes, MFI | Evolocumab (n = 34) | 38397±8768 | 35252±6095 | -3145 (-5406 to -885) | -1489 (-4620 to 1643) | 0.693 |
|  | Placebo (n = 37) | 38581±7813 | 36924±4732 | -1657 (-3824 to 510) |  |  |
| CD11b – non-classical monocytes, MFI | Evolocumab (n = 34) | 7563±1780 | 7661±2057 | 98 (-451 to 647) | 262 (-498 to 1023) | 0.740 |
|  | Placebo (n = 37) | 7922±1712 | 7758±1999 | -164 (-691 to 362) |  |  |

Differences in change between the treatment groups (evolocumab-treated patients, n = 34; placebo-treated patients, n = 37) were assessed using linear mixed-effects models, including time, treatment group, and their interaction as fixed effects, with a random intercept for participant. P-values were adjusted for multiple testing using the Benjamini–Hochberg procedure with a false discovery rate of 5%.

Abbreviations: CD11b, cluster of differentiation 11b; MFI, median fluorescence intensity

## Supplementary table 3. Change in cytokine production capacity of peripheral blood mononuclear cells by treatment group (n = 65)

| **Parameter** | **Treatment group** | **Baseline**  **mean±SD** | **Follow-up**  **mean±SD** | **Mean change**  **(95% CI)** | **Mean difference in change**  **(95%CI) vs. placebo** | **P_adj_ value** |
| --- | --- | --- | --- | --- | --- | --- |
| TNFα, pg/mL | Evolocumab (n = 32) | 153±222 | 201±228 | 48 (-44 to 140) | 58 (-71 to 187) | 0.466 |
|  | Placebo (n = 33) | 147±169 | 137±111 | -10 (-102 to 81) |  |  |
| IL-1β, pg/mL | Evolocumab (n = 32) | 2953±2212 | 3431±1875 | 478 (-240 to 1196) | 865 (-143 to 1873) | 0.434 |
|  | Placebo (n = 33) | 3136±2023 | 2749±1728 | -387 (-1094 to 320) |  |  |
| IL-6, pg/mL | Evolocumab (n = 32) | 7348±4793 | 10557±6531 | 3209 (661 to 5759) | 2463 (-1113 to 6039) | 0.434 |
|  | Placebo (n = 33) | 10012±6872 | 10758±6714 | 746 (-1763 to 3255) |  |  |
| IL-1Ra, pg/mL | Evolocumab (n = 32) | 14835±8472 | 18536±9501 | 3701 (64 to 7337) | -979 (-6082 to 4124) | 0.703 |
|  | Placebo (n = 33) | 14157±8190 | 18836±9726 | 4680 (1099 to 8260) |  |  |
| IL-10, pg/mL | Evolocumab (n = 32) | 379±461 | 629±490 | 250 (90 to 409) | 123 (-101 to 347) | 0.459 |
|  | Placebo (n = 33) | 393±354 | 519±408 | 127 (-30 to 284) |  |  |

Differences in change between the treatment groups (evolocumab-treated patients, n = 34; placebo-treated patients, n = 37) were assessed using linear mixed-effects models, including time, treatment group, and their interaction as fixed effects, with a random intercept for participant. P-values were adjusted for multiple testing using the Benjamini–Hochberg procedure with a false discovery rate of 5%.

Abbreviations: IL, interleukin; IL-1Ra, interleukin-1 receptor antagonist; TNF, tumour necrosis factor

## Supplementary table 4. Change in monocyte phenotype by infarct type (n = 71)

| **Parameter** | **Treatment group** | **Baseline**  **mean±SD** | **Follow-up**  **mean±SD** | **Mean change**  **(95% CI)** | **Mean difference in change**  **(95%CI) vs. placebo** | **P_adj_ value** |
| --- | --- | --- | --- | --- | --- | --- |
| Classical monocytes, % | STEMI (n = 24) | 78.6±8.6 | 74.3±5.8 | -4.4 (-8.3 to -0.4) | -4.4 (-9.3 to 0.4) | 0.222 |
|  | NSTEMI (n = 47) | 77.0±9.6 | 77.0±7.5 | 0.1 (-2.8 to 2.9) |  |  |
| Intermediate monocytes, % | STEMI (n = 24) | 7.0±3.7 | 7.6±3.4 | 0.6 (-0.8 to 2.0) | 0.8 (-0.9 to 2.5) | 0.537 |
|  | NSTEMI (n = 47) | 7.2±3.4 | 7.0±3.2 | -0.2 (-1.2 to 0.8) |  |  |
| Non-classical monocytes, % | STEMI (n = 24) | 4.5±2.5 | 8.2±4.2 | 3.6 (2.2 to 5.1) | 2.7 (0.9 to 4.6) | 0.024 |
|  | NSTEMI (n = 47) | 5.7±2.9 | 6.6±3.3 | 0.9 (-0.2 to 2.0) |  |  |
| CD11b – classical monocytes, MFI | STEMI (n = 24) | 56035±6112 | 56978±5289 | 943 (-1238 to 3125) | 609 (-2072 to 3291) | 0.651 |
|  | NSTEMI (n = 47) | 57553±8460 | 57887±7702 | 334 (-1225 to 1893) |  |  |
| CD11b – intermediate monocytes, MFI | STEMI (n = 24) | 40353±8043 | 36872±4316 | -3480 (-6169 to -792) | -1678 (-4982 to 1627) | 0.537 |
|  | NSTEMI (n = 47) | 37543±8238 | 35740±5958 | -1802 (-3724 to 119) |  |  |
| CD11b – non-classical monocytes, MFI | STEMI (n = 24) | 8042±1621 | 7808±1747 | -235 (-888 to 418) | -296 (-1099 to 507) | 0.557 |
|  | NSTEMI (n = 47) | 7601±1799 | 7662±2152 | 61 (-405 to 528) |  |  |

Differences in change between patients with STEMI (n = 24) and NSTEMI (n = 47) were assessed using linear mixed-effects models, including time, infarct type, and their interaction as fixed effects, with a random intercept for participant. P-values were adjusted for multiple testing using the Benjamini–Hochberg procedure with a false discovery rate of 5%.

Abbreviations: CD11b, cluster of differentiation 11b; MFI, median fluorescence intensity; NSTEMI, non-ST elevation myocardial infarction; STEMI, ST-elevation myocardial infarction

## Supplementary table 5. Change in cytokine production capacity of peripheral blood mononuclear cells by infarct type (n = 65)

| **Parameter** | **Treatment group** | **Baseline**  **mean±SD** | **Follow-up**  **mean±SD** | **Mean change**  **(95% CI)** | **Mean difference in change**  **(95%CI) vs. placebo** | **P_adj_ value** |
| --- | --- | --- | --- | --- | --- | --- |
| TNFα, pg/mL | STEMI (n = 23) | 95±123 | 114±107 | 19 (-90 to 128) | 0 (-135 to 136) | 0.998 |
|  | NSTEMI (n = 42) | 180±221 | 199±205 | 19 (-62 to 99) |  |  |
| IL-1β, pg/mL | STEMI (n = 23) | 2463±1816 | 2764±2061 | 301 (-562 to 1163) | 405 (-668 to 1478) | 0.998 |
|  | NSTEMI (n = 42) | 3365±2201 | 3260±1674 | -105 (-743 to 534) |  |  |
| IL-6, pg/mL | STEMI (n = 23) | 6558±4509 | 8641±6170 | 2084 (-966 to 5133) | 193 (-3601 to 3987) | 0.998 |
|  | NSTEMI (n = 42) | 9874±6492 | 11764±6594 | 1890 (-367 to 4147) |  |  |
| IL-1Ra, pg/mL | STEMI (n = 23) | 11986±6806 | 17827±9315 | 5841 (1577 to 10104) | 2542 (-2761 to 7846) | 0.998 |
|  | NSTEMI (n = 42) | 15862±8746 | 19160±9743 | 3298 (143 6453) |  |  |
| IL-10, pg/mL | STEMI (n = 23) | 244±291 | 458±489 | 214 (24 to 403) | 41 (-195 to 277) | 0.998 |
|  | NSTEMI (n = 42) | 464±443 | 636±420 | 173 (32 to 313) |  |  |

Differences in change between patients with STEMI (n = 23) and NSTEMI (n = 42) were assessed using linear mixed-effects models, including time, infarct type, and their interaction as fixed effects, with a random intercept for participant. P-values were adjusted for multiple testing using the Benjamini–Hochberg procedure with a false discovery rate of 5%.

Abbreviations: IL, interleukin; IL-1Ra, interleukin-1 receptor antagonist; NSTEMI, non-ST elevation myocardial infarction; TNF, tumour necrosis factor; STEMI, ST-elevation myocardial infarction
